# Prevalence, risk factors, and impact of lung Cancer on outcomes of idiopathic pulmonary fibrosis: a study from the Middle East

**DOI:** 10.1186/s40248-018-0150-7

**Published:** 2018-10-03

**Authors:** Sherif Mohamed, Hassan Bayoumi, Nashwa Abd El-Aziz, Ehab Mousa, Yasser Gamal

**Affiliations:** 1 Department of Chest Diseases and Tuberculosis, Faculty of Medicine, Assiut University, Assiut, 71516 Egypt; 2 Department of Medical Oncology, South Egypt Cancer Institute, Assiut University, Assiut, Egypt; 3 Department of Radiology, Faculty of Medicine, Assiut University, Assiut, Egypt; 4 Department of Pathology, Faculty of Medicine, Assiut University, Assiut, 71516 Egypt

**Keywords:** Prevalence, Impact, Lung cancer, Idiopathic pulmonary fibrosis, Survival, Upper Egypt

## Abstract

**Background:**

No studies have addressed the impact of lung cancer (LC) on prognosis of patients with idiopathic pulmonary fibrosis (IPF) in Upper Egypt. We aimed to evaluate the prevalence and risk factors for LC among IPF patients and its impact on their outcomes and survival in Upper Egypt.

**Methods:**

A total of 246 patients with IPF who had complete clinical and follow up data were reviewed. They were categorized into 2 groups: 34 patients with biopsy-proven LC and IPF (LC-IPF) and 212 patients with IPF only (IPF). Survival and clinical characteristics of the two groups were compared.

**Results:**

Prevalence of LC was 13.8%. Pack/years was the most significant predictor for LC development in IPF (Odds ratio; 3.225, CI 1.257–1.669, *p* = 0.001). Survival in patients with LC-IPF was significantly worse than in patients with IPF without LC; median survival, 35 months vs 55 months; *p* = 0.000. LC accompanying IPF was one of the most significant independent predictors of survival in IPF patients (Hazard ratio 5.431, CI 2.186–13.492, *p* = 0.000). Mortality in LC-IPF patients was mainly due to LC progression in 36% and LC therapy-related complications in 22%.

**Conclusions:**

Prevalence of LC in IPF patients was 13.8%. Lung cancer has significant impacts on patients with IPF in Upper Egypt, in terms of clinical outcomes and survival. Smoking is the most significant independent predictor of LC development in IPF patients. A poorer survival was observed for patients with IPF developing LC, mainly due to LC progression, and to complications of its therapies. Further prospective, multicenter and larger studies are warranted.

## Background

Idiopathic pulmonary fibrosis (IPF) is the most common of the idiopathic interstitial pneumonias and carries the worst prognosis [1]. Despite that several clinicopathologic and radiographic variables have been shown to correlate with survival in patients with IPF [2], the development of lung cancer (LC) among those patients further worsens their prognosis [3, 4].

Previous studies have addressed the clinical risk factors associated with LC development in IPF patients [3, 5–7]. A higher incidence of LC has been described in older male smokers and in patients with combined pulmonary fibrosis and emphysema (CPFE) [3, 5, 7, 8]. However, tumor location in patients with both LC and IPF (LC-IPF) differs among studies [3–7]. Furthermore, there is no consensus regarding the most prevalent histologic type of LC-IPF [6–10].

Previous studies had compared LC profiles in patients with and without IPF [3–10]. However, these studies included only populations from Asian [3, 6–9] and Western [4, 5, 10] countries. There is considerable lack of such studies in the Egyptian and/or Middle East populations. Ethnical differences could exist [11–13]. Therefore, in the current study, we aimed to evaluate the prevalence and risk factors for LC among patients with IPF and to address possible impact for LC on clinical outcomes and long-term survival of IPF patients in Upper Egypt.

## Methods

### Study design and population

Assiut University Hospital (AUH) and South Egypt Cancer Institute (SECI) are two large tertiary hospitals serving a large number of populations at Upper Egypt.

A systematic search of the patient database at the Chest Department of AUH revealed 267 patients who fulfilled the international guidelines on the diagnosis and management of IPF [1], during the period of July 1, 2012, to July 31, 2017. Among these 267 patients, we selected 246 patients diagnosed with IPF that were then followed up regularly at our Department according to a prospective protocol for follow up of IPF patients, including clinical management, annual high resolution computed tomography (HRCT) scanning, and regular pulmonary function testing (PFT). The remaining 21 patients were excluded due to incomplete follow up data.

These 246 cases were categorized in two groups: (1) the group of 34 patients who had (at the time of diagnosis of IPF) or developed LC (biopsy-proven) during the follow up period, named the LC-IPF group. Once diagnosed with LC, the patients were referred to the Medical Oncology Department, SECI, for further oncologic management, and (2) the group of 212 patients with IPF only (IPF group). The 2 groups were compared for clinical features, outcomes, and survival analysis. The later was carried out in all cases from the time of IPF diagnosis.

### Data collection and diagnostic criteria

The medical records of all IPF patients were reviewed to obtain data regarding age, smoking history, the method used to diagnose IPF, time of IPF diagnosis, PFT, HRCT findings, comorbidities, follow up duration, and outcome. Pack/years value was calculated by multiplying the number of packs of cigarettes smoked per day by the number of years the person has smoked [14]. The findings of HRCT scans were classified as reticular, honeycomb, ground-glass opacities, and nodular patterns. An acute exacerbation of IPF (AEIPF) was defined as acute respiratory worsening for which a cause could not be identified and meeting all criteria for as proposed by the international agreement [15], and their updates [16]. Patients diagnosed as combined pulmonary fibrosis and emphysema (CPFE) were not enrolled in the study.

For the LC-IPF group, the time of diagnosis and location of LC including the IPF-associated area were analyzed. All cases of LC were biopsy-proven. Lung cancers were classified according to the World Health Organization classification. Staging of LC has been established by the TNM system current at the time of diagnosis. Side effects of treatments were assessed using the National Cancer Institute Common Terminology Criteria for Adverse Events (CTCAE, version 4.0) [17]. Evaluation of tumor response to chemotherapy was assessed according to the Response Evaluation Criteria in Solid Tumors (RECIST) criteria; version 1.1 [18]. Mortality related to either surgical intervention and/or oncologic treatment was defined as death occurring within 30 days of treatment. Date of death was verified using hospital records, local death registry review, and review of subsequent patient visits.

The study was approved by the Ethical Committee of AUH and SECI.

### Statistical analysis

Patient demographics were compared using the unpaired t-test for continuous variables and the Pearson’s *χ*^2^ test for categorical variables. Logistic regression analysis was performed to assess the risk of LC in IPF patients. Survival was estimated using Kaplan-Meier curves. The survival rates of patients in the IPF only group and the IPF with lung cancer (LC-IPF) group were compared using a log-rank test. Cox’s proportional hazards regression analysis was used to identify significant variables that affect the survival of IPF patients and to estimate hazard ratios (HR) and 95% confidence intervals (CI) for predictors of survival. All tests were two-tailed, and *p* < 0.05 were considered statistically significant. SPSS software Version 22.0 (SPSS Inc., Chicago, IL, USA) was used for all statistical analyses.

## Results

### Patient characteristics

The prevalence of LC in IPF patients was 13.8%. Table 1 summarizes the clinical findings in IPF patients grouped according to the presence or absence of LC. The mean age at diagnosis of IPF was 52.4 ± 8.2 years, and 69.5% of IPF patients were males. When the 2 groups were compared, there were significant differences in age, smoking status, pack/years, forced vital capacity (FVC), forced expiratory volume in 1 s (FEV_1_), diffusing capacity of the lung for carbon monoxide (D_LCO_), and AEIPF.

**Table 1:** Demographics of patients with idiopathic pulmonary fibrosis in the 2 study groups

Characteristic	All IPF	IPF and LC	IPF only	*P*
Subjects No. (%)	246 (100)	34 (13.8)	212 (86.2)	0.000
Age,± SD, y	52.4 ± 8.2	56.6 ± 10.0	47.7 ± 10.2	0.002
Gender No. (%)				0.107
Male	171 (69.5)	28(82.3)	143 (67.4)	
Female	75 (30.5)	6 (17.7)	69 (32.6)	
Smoking status				0.033
Current	180 (73.2)	22 (64.6)	158 (74.4)	
Past	31 (12.6)	4 (11.8)	27 (12.8)	
Never	35 (14.2)	8 (23.6)	27 (12.8)	
Pack/years	33.4 ± 16.7	37.2 ± 21.7	30.7 ± 13.1	0.016
FVC (% pred)	80.8 ± 2.9	85.9 ± 2.7	77.1 ± 3.8	0.000
FEV_1_(% pred)	82.3 ± 5.0	87.0 ± 4.8	76.9 ± 5.9	0.010
TLC (% pred)	69.2 ± 10.1	70.1 ± 12.8	67.8 ± 9.3	0.206
D_LCO_ (% pred)	60.2 ± 8.3	57.1 ± 10.4	62.6 ± 9.5	0.000
Diagnostic method				0.021
Clinical	218 (88.6)	26 (76.5)	192 (90.6)	
Surgical	28 (11.4)	8 (23.5)	20 (9.4)	
AEIPF	87 (35.3)	18 (53)	69 (32.5)	0.032

Data are presented as mean ± SD, percentage or median (min-max) unless otherwise indicated. % pred, percentage of the predicted value; FVC; forced vital capacity, FEV_1_; forced expiratory volume in 1 s, TLC; total lung capacity, D_LCO_; diffusing capacity of the lung for carbon monoxide, AEIPF; acute exacerbations of idiopathic pulmonary fibrosis

### HRCT and clinicopathologic findings in LC-IPF patients

Among the 34 patients with LC-IPF, 12 (35.3%) were diagnosed as having primary pulmonary LC at the same time of IPF diagnosis. The other 22 (64.7%) patients developed LC 19.4 ± 16.8 months (median, 32 months; range, 3.2–78.4 months) after diagnosis of IPF during the follow up period. The cumulative incidence of LC at 1 and 3 years was 37.2 and 62.5%, respectively.

Most of the cancerous lesions were located in peripheral areas of the lung (73.5%). Two-thirds (67.6%) of the lesions were in the lower lobes. Also, two-thirds of the cancerous lesions were located in a fibrotic background (IPF-associated areas) in the same patients. Squamous cell carcinoma (44%) and adenocarcinoma (41%) were the most common histological findings in LC-IPF patients.

Eight (23.3%) patients had early stage I lung cancer. Five (15%), 9(26.4%), and 12(35.3%) patients had stage II, III, and IV, respectively. Table 2 shows the radiological and clinicopathological findings in LC-IPF patients.

**Table 2:** HRCT findings, histopathology and stages of LC in patients with LC-IPF (*n* = 34)

Characteristic	No. (%)
Time of LC diagnosis	
At the time of IPF diagnosis	12 (35.3)
After IPF diagnosis	22 (64.7)
Location	
Central^a^	9 (26.5)
Peripheral	25 (73.5)
Lobes of the lung	
Upper lobes	10 (29.5)
Middle lobe	1 (2.9)
Lower lobes	23 (67.6)
HRCT background	
Fibrotic background^b^	23 (67.6)
Nonfibrotic background	11 (32.4)
Histopathology	
Squamous cell carcinoma	15 (44)
Adenocarcinoma	14 (41)
Adenosquamous carcinoma	2 (6)
large cell carcinoma	2 (6)
Undifferentiated carcinoma	1 (3)
Stage	
I	8 (23.3)
II	5 (15.0)
III	9 (26.4)
IV	12 (35.3)

*LC-IPF* combined lung cancer and idiopathic pulmonary fibrosis, *HRCT* high-resolution computed tomography

^a^Central; the tumor was considered central when located in an area 3 cm far from the pleura

^b^Fibrotic background; includes reticular distortion, ground-glass opacity, and honeycombing

### Risk factor analysis of lung cancer

To assess possible risk factors for the development of LC in IPF patients, variables including age, gender, smoking, pack/years, and D_LCO_ were analyzed. Multivariate regression after adjustment for significant factors revealed that pack/years and male gender were significant independent predictors of LC in IPF patients (Table 3).

**Table 3:** Multivariate analysis of risk factors for predicting the development of lung cancer in idiopathic pulmonary fibrosis

Variables	OR	95% CI	*P*-
Gender, male	1.089	0.014–0.579	0.011
Age,	0.677	0.214–9.757	0.707
Smoking	0.411	0.378–12.869	0.279
Pack/year	3.225	1.257–1.669	0.001
D_LCO_	0.849	1.016–1.138	0.062

*CI* confidence interval, *OR* odds ratio, *D*_*LCO*_ diffusing capacity of the lung for carbon monoxide

### Therapies for LC and their complications

Out of the 34 patients with LC-IPF, 4 underwent surgery, while 5,2,13, and 10 received radiotherapy, radio-chemotherapy, chemotherapy, and best supportive care, respectively. The decisions for different therapeutic modalities were undertaken by a multidisciplinary team, taking into consideration the underlying IPF. A total of 17/24 (71%) patients experienced therapy-related complications. Surgery for early LCs consisted of four lobectomies. Three patients (75%) had complications, mainly pneumonia. Estimated 30-day mortality was 25%. Complications were reported in 3/5 (60%), 1/2(50%), and 10/13(77%) of patients who underwent radiotherapy, radio-chemotherapy, and chemotherapy, respectively. Those who received radio- or radio-chemotherapy were among the 11 patients with non-fibrotic lung pathology. Among 13 patients treated with chemotherapy only, 5 (38.5%) patients died with a median survival of 7 months (range, 3–22 months). Table 4 details these results. Patients with LC-IPF did not receive antifibrotic therapy (pirfenidone/nintedanib) for their IPF.

**Table 4:** Therapy-related complications and acute exacerbations of IPF in LC-IPF patients (*n* = 34)

Complications	%	AEIPF, No. (%)	Mortality of AEIPF, No. (%)
Surgery-related complications (*n* = 3/4)	75%	2/4 (50)	1/2 (50)
Respiratory failure	25%		
Pneumonia	50%		
Empyema	25%		
Myocardial infarction	25%		
Radiotherapy-related complications (*n* = 3/5)	60%	2/5 (40)	0/2 (0)
Radiation pneumonitis	33%		
Pulmonary infections	33%		
Respiratory failure	17%		
Myocardial infarction	17%		
Radio-chemotherapy-related complications (*n* = 1/2)	50%	0/2 (0)	0/1 (0)
Radiation pneumonitis	50%		
Chemotherapy-related complications (*n* = 10/13)	77%	8/13 (61.5)	5/8 (62.5)
Pancytopenia	30%		
Pneumonia	40%		
Respiratory failure	20%		
Cardiovascular failure	25%		
Total	71%	12/24 (50)	6/12 (50)

*LC* lung cancer, *IPF* idiopathic pulmonary fibrosis, *AEIPF* acute exacerbation of idiopathic pulmonary fibrosis

### Survival analysis

**Fig. 1:**
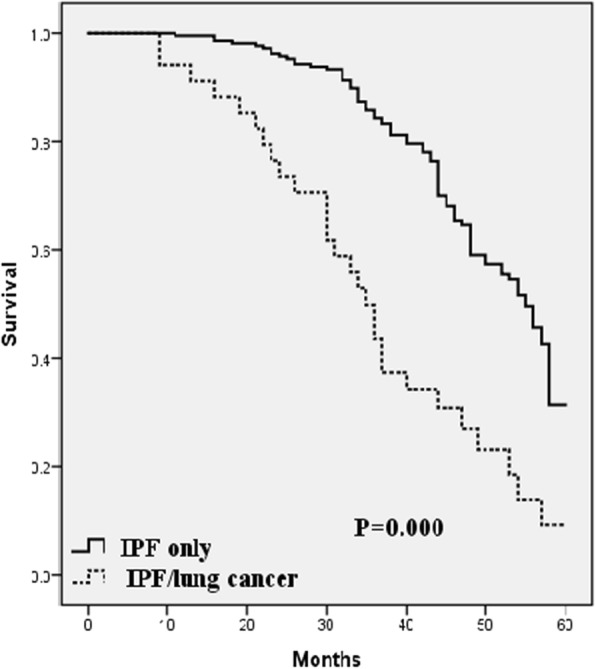
Comparison of survival between idiopathic pulmonary fibrosis with lung cancer (LC-IPF) patients and idiopathic pulmonary fibrosis (IPF) only patients; Kaplan-Meier survival curve

Among the 212 patients with IPF without LC, 93(43.8%) died, median survival was 55.0 months, while in the group of patients with LC-IPF, 28(82.3%) patients died, median survival was 35.0 months. Survival time was significantly different between the 2 groups (*p* = 0.000, log-rank test). One- and 3-year survivals among the two groups were 90% and 52% in the LC-IPF group and 98 and 83% in the IPF group, respectively. Survival curves of patients with IPF with and without LC are displayed in Fig. 1.

Lung cancer accompanying IPF was one of the most significant independent predictors of survival in IPF patients (HR 5.431, CI 2.186–13.492, *p* = 0.000). The other significant predictor was smoking (HR 2.114, CI 1.370–5.118, *p* = 0.004). Table 5 summarizes the HR of each variable in the total sample of patients with IPF.

**Table 5:** Cox regression analysis for predictors of survival in patients with idiopathic pulmonary fibrosis

Variables	Hazard Ratio	95% CI	*P*
Gender	0.615	0.318–1.192	0.150
Age	1.015	0.991–1.040	0.224
Smoking	2.114	1.370–5.118	0.004
Pack/year	0.998	0.968–1.029	0.880
FVC (% pred)	1.018	0.964–1.001	0.057
FEV_1_(% pred)	1.008	0.957–1.062	0.766
TLC (% pred)	0.702	0.956–1.014	0.298
D_LCO_ (% pred)	1.148	0.319–4.966	0.067
Lung cancer	5.431	2.186–13.492	0.000

*CI* confidence interval, *FVC* forced vital capacity, *%* pred; percentage of predicted value, *FEV*_*1*_ forced expiratory volume in 1 s, *TLC* total lung capacity, *D*_*LCO*_ diffusing capacity of the lung for carbon monoxide

In the LC-IPF group, six (22%) patients died of lethal complications related to LC therapies. Four (14%) died of AEIPF. Ten (36%) patients died of LC progression, and eight (28%) patients died of respiratory failure related to IPF.

### Progression of IPF

Median follow up duration was 32 months (range, 1.0–60.0 months) in the LC-IPF group and 42.6 months (range, 6.0–98.2 months) in the IPF group. During the follow up period, 24 patients among 34 with LC (70.6%) and 122 among 212 without LC (57.5%) experienced IPF progression. Median time-to-disease progression was 24 months in the LC-IPF group and 27 months in the IPF group. There was not a significant difference in progression-free survival between the two groups; *p* = 0.087. After adjusting for age, gender, smoking status, % predicted D_LCO_, and % predicted FVC, there was no difference (HR 1.359; 95% CI, 0.774–2.338; *p* = 0.131).

### AE of IPF

A total of 87 patients (35.3%) experienced AEIPF during the study period, with a significant difference between the 2 groups; 18/34 (53%) were in the LC-IPF group, and 69/212 (32.5%) were in the IPF group, *p* = 0.032, respectively. (Table 1) Mortality for AE was 55.6% (10 of 18) and 71% (49 of 69) in LC-IPF and IPF-only, respectively. Notably, in patients with LC-IPF, AEs were triggered by therapy for LC in 50% of cases (12/24); two surgery (of four patients, 50%), two radiotherapies (of five patients, 40%), and 8 chemotherapies (of 13 patients, 61.5%). Mortality due to treatment-related AEs was reported in 50% (5/10) of cases. Table 4 shows AEIPF related to LC therapies.

## Discussion

To the best of our knowledge, this is the first study that addresses the impact of LC on IPF patients in the Middle East and Egypt. Results showed that LC has significant impacts on clinical outcomes and survival of patients with IPF in Upper Egypt. Demographic features of patients with IPF and/or LC could be different in our locality. Mohamed and Ibrahim [11], in their study of 568 patients with IPF in Upper Egypt, concluded that IPF has a different age and sex distribution in Upper Egypt, compared to international data. It was observed that 43% of patients with IPF developed their disease before the age of 50, with a mean age of 48.6 ± 12.9 years at the time of diagnosis [12]. In a recent study for primary LC in Upper Egypt, it was observed that the incidence of adenocarcinoma surpassed that of squamous cell carcinoma, and it was common that the patients presented with their illness at later stages [13].

Our data revealed that the prevalence of LC in IPF patients is 13.8% in all (8.9% in LC onset during the IPF follow up). The cumulative incidence of LC among IPF patients at 1 and 3 years was 37.2 and 62.5%, respectively. Our results are consistent with the worldwide reported prevalence range of 4.8 to 48% [3–5, 7]. Moreover, these data represent the third report; after those of Ozawa et al. [19] and Tomassetti et al [4], documenting that cumulative incidence of LC increases remarkably overtime after the initial diagnosis of IPF. Furthermore, these data confirm the crucial importance of a prospective protocol for follow up of IPF patients with annual HRCT scanning.

It has been observed that LC was more common among IPF patients who are older, males, and smokers. These findings are consistent with those of previous reports enrolling different populations [3–7, 9, 10]. For these reasons, we think that those particular populations with IPF should receive intensified follow up protocols for the development of LC. Despite that tumor location in LC-IPF patients was different among studies [3–7], results of the current study are in agreement with the majority of other studies of LC in IPF [3–10]. Most of the cancerous lesions were peripherally located, in the lower lobes, and in IPF-associated lesions. These findings add more support to the theory that the inflammatory process is associated with bronchiolar metaplasia in the pathogenesis of LC [20]. Again, we believe that those populations deserve more attention during their follow up protocols. It is necessary to raise awareness of LC risk among patients with IPF.

Similarly, there was no consensus regarding the most prevalent histologic type of LC-IPF [3–10]. Squamous cell carcinoma was the most common histological type encountered by some authors [4, 5, 10], while adenocarcinoma was encountered by others [21, 22]. Squamous cell carcinoma and adenocarcinoma were found in 44 and 41% of our LC-IPF cohort, respectively. In a recent systemic review, Wang et al. concluded that adenocarcinoma and squamous cell carcinoma are the most common types in IPF-associated LC patients [23].

Considering the clinical, radiologic, and pathologic findings of our cohort together may support the current hypothesis that considers IPF as a disease of premature aging with several links to LC [4, 5, 8, 24]. However, the pathogenesis of combined IPF and LC is not so simple. Recently, it has been proposed that perturbed signaling pathways, epigenetic and genetic changes, oxidative stress, and fibroblast growth factor receptor (FGFR) signaling pathways are all thought to be involved in the pathogenesis of IPF-associated LC [23].

Previous studies have highlighted the clinical risk factors associated with LC development in IPF patients [3–7, 25]. It was consistently shown that elderly male IPF patients with a history of smoking are more likely to develop LC. In this context, we observed that pack/years and male gender were significant independent predictors of LC in IPF patients. A study from England suggested that the incidence of LC is significantly increased in IPF patients compared to the general population, and that smoking is an independent predictor of LC development [26].

Survival analysis of our cohort showed interesting results. Survival time was significantly different between LC-IPF and IPF only (*p* = 0.000). LC accompanying IPF was one of the most significant independent predictors of survival in IPF patients (HR 5.431, CI 2.186–13.492, *p* = 0.000).

Notably, the difference in mortality seen in our study was not attributable to worsening of pulmonary fibrosis, but mainly to both LC progression and complications of LC therapies. Similar to the findings observed by Tomassetti et al. [4], this is the second report that confirms such significant findings.

Our results are in strong agreement with previous reports [3–7]. Lee and coworkers [3] concluded that LC was the most predictor for mortality among IPF patients (HR 2.441, CI 1.373–4.339; *p* = 0.002), while Tomassetti et al [4], observed a statistically significant difference in survival of patients with LC-IPF compared with IPF only, with an adjusted HR of 7 (95% CI, 3.81–12.90; *p* = 0.001).

The majority of deaths among LC-IPF patients seen in the current study was not due to worsening of pulmonary fibrosis, but to both LC progression and complications of LC treatment; in 36 and 22% of patients, respectively. Moreover, it was observed that 50% of AEs in patients with LC were triggered by therapies for LC. These risks make the decisions about LC management in IPF, despite undertaken by a multidisciplinary team, still challenging to the clinician.

Furthermore, two-thirds of patients with LC-IPF (23/34, 67.6%) had advanced cancer and received either chemotherapy (with a high rate of complications) or best supportive care. Despite improvements in our understanding of pathogenetic mechanisms for the development of LC among IPF patients [23], the current treatment strategies for combined IPF and LC, including medications and surgery are still controversial, complex and thorny [4, 23, 27].

Several studies had addressed the impact of surgical resection of LC in patients with IPF. Early postoperative mortality ranged from 0 to 18.2% and postoperative morbidity ranged from 7.1 to 40.7% [27, 28]. Notably, we reported surgery-related complications in 75% of operated patients, mainly pneumonia. Kreuter and colleagues reported an incidence of 67% for surgery-related complications and a 30-day mortality of 25% among the operated patients with LC-IPF [10].

This high rate of surgery-related complications, again highlights the importance of annual HRCT for patients with IPF, for early detection of LC which might give the patient better surgical therapeutic options, hence better outcomes.

There are only sparse data concerning radiotherapy in LC with IPF [10]. Despite those patients who received radio-, or radio-chemotherapy had non-fibrotic ground in HRCT still, the reported rates of radiation pneumonitis and pulmonary infections were high, but with no impact on mortality. Local tumor ablation might be an attractive option in some of those severely pulmonary compromised patients, while it has been used safely in patients with severe emphysema [29]. Disappointingly, it seems that chemotherapy is not a safe therapeutic option for patients with LC-IPF. Chemotherapy-related toxicities were reported in 77% of our cohort, mainly pneumonia, and pancytopenia. Previous studies reported chemotherapy-related toxicities in 63% [10] and 50% [4] of patients with LC-IPF.

Acute exacerbations have significant impact on IPF patients, particularly those with LC-IPF, with considerable morbidity and mortality. In the literature, the incidence of treatment-related AEs in patients with LC-IPF ranged between 12.5 and 30% and mortality of treatment-related AE ranged between 9 and 16% [30, 31]. However, our results revealed a higher incidence of treatment-related AEs (50%) and their related mortality (50%). Careful monitoring of patients for these AEs, and if possible, their prediction [32], are warranted.

Despite the current study is the first one in Egypt and the Middle East that describes the impact of LC on IPF in a unique group of populations, yet it is to be considered that the inherent disadvantage of being a retrospective study, together with its single-center design, and the relatively limited number of patients are important limitations. The impact of LC on IPF and the optimal management of LC in patients with IPF needs to be addressed in larger prospective multicenter studies. Finally, our data from Upper Egypt are generally similar to those previously reported from Western and Asian populations.

## Conclusions

Lung cancer has significant impact on patients with IPF in Upper Egypt, in terms of clinical outcomes and survival. Smoking and male gender were significant independent predictors of lung cancer development in IPF patients. A poorer survival rate was observed in patients with IPF developing LC, mainly due to LC progression, and to complications of its treatments. Chemotherapy and surgery had a high incidence of fatal complications. Careful monitoring of the patients for these complications is needed. Further prospective, multicenter and larger studies are warranted.

## Abbreviations

AEIPF: Acute exacerbation of idiopathic pulmonary fibrosis
CPFE: Combined pulmonary fibrosis and emphysema
CTCAE: Common Terminology Criteria for Adverse Events
D_LCO_: Diffusing capacity of the lung for carbon monoxide
FEV_1_: Forced expiratory volume in 1 s
FGFR: Fibroblast growth factor receptor
FVC: Forced vital capacity
HR: Hazard ratio
HRCT: High resolution computed tomography
IPF: Idiopathic pulmonary fibrosis
LC: Lung cancer
LC-IPF: Combined lung cancer and idiopathic pulmonary fibrosis
OR: Odds ratio
PFT: Pulmonary function tests
RECIST: Response evaluation criteria in solid tumors
TLC: Total lung capacity
TNM: Tumor, node, metastasis
WHO: World Health Organization
